# Demands of eicosapentaenoic acid (EPA) in *Daphnia*: are they dependent on body size?

**DOI:** 10.1007/s00442-016-3675-5

**Published:** 2016-06-27

**Authors:** Anna B. Sikora, Thomas Petzoldt, Piotr Dawidowicz, Eric von Elert

**Affiliations:** 1 Department of Hydrobiology, Faculty of Biology, Biological and Chemical Research Center, University of Warsaw, Żwirki i Wigury 101, 02-089 Warsaw, Poland; 2 Institute of Hydrobiology, Technische Universität Dresden, Helmholtzstrasse 10, 01069 Dresden, Germany; 3 Aquatic Chemical Ecology, Cologne Biocenter, University of Cologne, Zülpicherstrasse 47b, 50674 Cologne, Germany

**Keywords:** Body size, *Daphnia*, EPA, Polyunsaturated fatty acids, Threshold

## Abstract

Fatty acids contribute to the nutritional quality of the phytoplankton and, thus, play an important role in *Daphnia* nutrition. One of the polyunsaturated fatty acids (PUFAs)––eicosapentaenoic acid (EPA)––has been shown to predict carbon transfer between primary producers and consumers in lakes, suggesting that EPA limitation of *Daphnia* in nature is widespread. Although the demand for EPA must be covered by the diet, the demand of EPA in *Daphnia* that differ in body size has not been addressed yet. Here, we hypothesize that the demand for EPA in *Daphnia* is size-dependent and that bigger species have a higher EPA demand. To elucidate this, a growth experiment was conducted in which at 20 °C three *Daphnia* taxa (small-sized *D. longispina* complex, medium-sized *D. pulicaria,* and large-bodied *D. magna*) were fed *Synechococcus elongatus* supplemented with cholesterol and increasing concentrations of EPA. In addition, fatty acid analyses of *Daphnia* were performed. Our results show that the saturation threshold for EPA-dependent growth increased with increasing body size. This increase in thresholds with body size may provide another mechanism contributing to the prevalence of small-bodied cladocera in warm habitats and to the midsummer decline of large cladocera in eutrophic water bodies.

## Introduction

Allometric relationships for many of the essential features of organisms are widely observed (Woodward et al. 2005). Body size of zooplankton affects directly and indirectly metabolic rate, feeding, respiration, growth rate, reproduction, and mortality (reviewed in Hart and Bychek 2011). In addition, the competitive ability of zooplankton is strongly correlated with zooplankton body size, and large-bodied species are competitively superior (Brooks and Dodson 1965).

Zooplankton species differ with respect to their elemental composition (C:P ratio). In line with the fact that *Daphnia* showed the lowest C:P ratio, *Daphnia* are assumed to be much more sensitive to a low P content in phytoplankton than other herbivorous zooplankton taxa (Hessen and Lyche 1991; Hessen 1992). However, the phosphorus demand is higher in large-bodied species due to their higher growth rate than in small-bodied species that grow slower (Sterner and Schulz 1998). Similar considerations among aquatic consumer taxa have led to the concept of threshold elemental ratios of carbon and phosphorus. These threshold ratios varied considerably among 41 investigated taxa, which could be attributed to interspecific differences in body C:P ratios and gross growth efficiencies of carbon (Frost et al. 2006).

Food quality is an important factor influencing the competitive ability of zooplankton (Moore et al. 1996; Iwabuchi and Urabe 2012). Polyunsaturated fatty acids (PUFAs) have been suggested to be a major determinant of the nutritional quality of algae (Ahlgren et al. 1990), which is explained by the fact that PUFAs regulate the fluidity of cell membranes (Hazel 1995) and that some PUFAs serve as precursors for hormones (e.g., prostaglandins) involved in the regulation of reproduction and many other metabolic processes in arthropods (Blomquist et al. 1991; Vance and Vance 2008). Some of the PUFAs, i.e., eicosapentaenoic acid (EPA; 20:5ω3), docosahexaenoic acid (DHA; 22:6ω3), arachidonic acid (ARA; 20:4 ω6), α-linolenic acid (ALA; 18:3ω3), and linoleic acid (LIN; 18:2ω6), are described as being essential fatty acids for zooplankton (Wacker and von Elert 2001; Smyntek et al. 2008). A limitation by these essential nutrients causes a reduction in somatic growth and reproduction (von Elert 2002, 2004; Becker and Boersma 2003; Ravet et al. 2003). Correlative studies have repeatedly provided evidence for an important role of EPA in determining the food quality of natural phytoplankton for *Daphnia*. In nature, *Daphnia* growth may be limited by the content of EPA in the diet (Müller-Navarra 1995; Müller-Navarra et al. 2000; Wacker and von Elert 2001; Hartwich et al. 2012). EPA not only potentially limits somatic growth, but is even more important for reproduction (Martin-Creuzburg et al. 2008, 2009; Ravet et al. 2012). This is confirmed by the observation that there is a higher allocation of EPA into eggs than into somatic tissue of *Daphnia* (Becker and Boersma 2005; Müller-Navarra 2006; Wacker and Martin-Creuzburg 2007; Sperfeld and Wacker 2012). The importance of EPA allocation into eggs in *Daphnia* is corroborated by the finding that the PUFA-content of the neonates depends on the diet consumed by mothers (Sperfeld and Wacker 2015). In line with this, maternal effects on the growth of *Daphnia* neonates have been demonstrated (Pajk et al. 2012; Sperfeld and Wacker 2015).

EPA can be synthesized by *Daphnia* from precursors (Schlechtriem et al. 2006)––e.g., linolenic acid (ALA) or docosahexaenoic acid (DHA), but the efficiency of this process might not be sufficient to maintain optimal growth (Weers et al. 1997; von Elert 2002). As only 2 % of all fatty acids can be synthesized de novo by *Daphnia* (Goulden and Place 1993), fatty acid demands have to be covered by the diet.

An approach to determine the demands for EPA is the calculation of EPA-saturation thresholds, which are defined as the minimal concentration of EPA above which the juvenile growth rate becomes saturated with respect to EPA (Sperfeld and Wacker 2011; Ravet et al. 2012). Threshold values of EPA vary with water temperature (Sperfeld and Wacker 2011), probably because animals need more EPA to maintain membrane fluidity at lower temperatures (Schlechtriem et al. 2006; Sperfeld and Wacker 2012). EPA thresholds also vary with the availability of other food components, such as sterols (cholesterol), e.g., the EPA threshold is lower when cholesterol availability is high (Sperfeld and Wacker 2011; Sperfeld et al. 2012, 2016). The metabolic requirements of essential fatty acids by zooplankton are expected to vary with the animals’ body size. In terms of food quantity, threshold food concentrations for zero growth in cladocerans decline with increasing body size (Gliwicz 1990), but thresholds for particular food components may not follow this pattern. Large-bodied species have been shown to be more vulnerable to temperature-related decreases in food quality of algae than small-bodied ones (Sikora et al. 2014). Therefore, we here hypothesized that the physiological demands for EPA might be higher in large-bodied zooplankton species compared with small-bodied ones.

The objective of this study was to test the hypothesis that the demand of a PUFA in a poikilothermic animal is size-dependent and that bigger species have a higher PUFA demand. We, therefore, ran growth experiments with four clones of three taxa, i.e., small-bodied *D. longispina* complex, medium-bodied *D. pulicaria,* and large-bodied *D. magna*, on a gradient of EPA ranging from 0 to 10 μg EPA mg C^−1^ and, subsequently, calculated thresholds for 50 % (=*K*_S_) and 75 % EPA saturation.

## Materials and methods

### Cultivation of algae and cyanobacterium

The green alga *Chlamydomonas klinobasis* (strain 56, culture collection of the Limnological Institute at the University of Konstanz, Germany) was used as a food for *Daphnia* before the experiment and in the control treatments. *C. klinobasis* was cultivated semi-continuously in 5-L batch cultures at 20 °C with a light intensity of 120 μmol m^−2^ s^−1^. Every second day 20 % of the culture was replaced by fresh, sterile cyano medium (von Elert and Jüttner 1997) with vitamins (thiamine hydrochloride 300 nmol L^−1^, biotin 2 nmol L^−1^, and cyanocobalamine––vitamin B12 0.4 nmol L^−1^).

The strain of *C. klinobasis* used here did not contain EPA (Von Elert and Stampfl 2000). The unicellular cyanobacterium *Synechococcus elongatus* (strain 89.79, Sammlung von Algenkulturen, Universität Göttingen, Germany) was grown in chemostats in Cyano medium (Von Elert and Jüttner 1997) at a dilution rate of 0.2 day^−1^ at 20 °C with a light intensity of 50 μmol m^−2^ s^−1^. *S. elongatus* was chosen for the experiments, because it is non-toxic and lacks sterols and PUFAs (von Elert and Wolffrom 2001). Carbon concentrations of the algal and cyanobacterial cultures were estimated from photometric light extinctions at 470 nm using previously established carbon-extinction regressions.

### Growth experiments

*Daphnia* were maintained in aged and filtered (0.45-μm filter) tap water at 20 °C with *C. klinobasis* at non-limiting food concentrations of 2 mg C L^−1^. Standardized growth experiments were carried out with four clones of the small-bodied species from the *D. longispina* complex (DlE, Dl4, Dh, and Dg), four clones of the medium-bodied *D. pulicaria* (DpBrA3, DpBrS, DpGr8, and DpGr49), and four clones of the large-bodied *D. magna* (IL-M1-12, IL-M1-8, FI-N26-8c, and FI-N47-20). These clones from the genus *Daphnia* were chosen to use animals of varying body size, but similar in environmental requirements (e.g., feeding regimes). Origin and body size of the experimental clones are given in Table 1.

**Table 1:** Origin and body size at first reproduction (mm) of the experimental *Daphnia* clones

Species	Clones	Origin	Body size (mm)	SE (mm)	Obtained from	Reference
*D. longispina* complex	*D. longispina* (DlE)	Lake Roś, Poland	1.43	0.02	T. Brzeziński	–
	*D. longispina* (Dl4)	Lake Roś, Poland	1.49	0.02	T. Brzeziński	–
	*D. hyalina* (Dh)	Lake Constance, Germany	1.59	0.02	D. Martin-Creuzburg	Stich and Lampert (1984)
	*D. galeata* (Dg)	Lake Constance, Germany	1.77	0.02	D. Martin-Creuzburg	Stich and Lampert (1984)
*D. pulicaria*	DpBrA3	Lake Brome, Canada	1.90	0.01	M. Ślusarczyk	Gélinas et al. (2007), Slusarczyk and Pietrzak (2008)
	DpBrS	Lake Brome, Canada	2.09	0.03	M. Ślusarczyk	Gélinas et al. (2007), Slusarczyk and Pietrzak (2008)
	DpGr8	Lake Greifen, Switzerland	2.28	0.04	P. Spaak	–
	DpGr49	Lake Greifen, Switzerland	2.31	0.03	P. Spaak	–
*D. magna*	IL-M1-12	Pond, Jerusalem, Israel	3.06	0.02	D. Ebert	Yampolsky et al. (2014)
	IL-M1-8	Pond, Jerusalem, Israel	3.18	0.02	D. Ebert	Yampolsky et al. (2014)
	FI-N26-8c	Lake Tvärminne, Finland	3.33	0.09	D. Ebert	Yampolsky et al. (2014)
	FI-N47-20	Lake Tvärminne, Finland	3.54	0.04	D. Ebert	Yampolsky et al. (2014)

Data are means ±1SE from 8 to 15 individuals

Growth experiments were initiated with neonates (<18 h old) from the second clutch of the clonal mothers. The experiments were carried out in 200 ml glass beakers containing ten individuals of *D. longispina* or *D. pulicaria* and in 250 ml glass beakers containing five individuals of *D. magna*. The concentration of alga or cyanobacterium was 2 mg C L^−1^ in all treatments. Seven experimental treatments were performed, in which *Daphnia* were fed with the cyanobacterium *S. elongatus* supplemented with 17.4 μg cholesterol mg C^−1^ and seven increasing volumes of eicosapentaenoic acid (EPA)-containing liposomes, which resulted in a dietary content ranging from 0 to 10 μg EPA mg C^−1^. To maintain equal concentrations of liposome in all food treatments, appropriate amounts of control liposomes were added in treatments with <10 μg EPA mg C^−1^. In three additional control treatments, the animals were fed with (1) the green alga *C. klinobasis*, (2) the cyanobacterium *S. elongatus* without any liposomes, and (3) *S. elongatus* with control liposomes at a concentration equivalent to the total concentration of cholesterol- and EPA-liposomes added to the food treatment with 10 μg EPA mg C^−1^. Each food and control treatment was carried out in triplicate. The animals were transferred daily into fresh food suspensions. The experiment was run at a constant temperature of 20 °C for 6 days. Somatic growth rate (*g*) was calculated according to the formula: $g \, = \, \left( {\ln \,W_{t} {-}\ln \,W_{ 0} } \right) \, \times \,t^{ - 1}$, where *W*_0_ is the initial dry mass of neonates, *W*_*t*_ is the weight of the individual at the end of experiment, and *t* is the duration of the experiment (Wacker and Von Elert 2001). For the determination of the initial dry mass, subsamples of ten individuals were used. The number of eggs in the brood chamber carried by *Daphnia* was counted under a dissecting microscope at the end of the experiment.

### Supplementation with fatty acids

*S. elongatus* was supplemented with EPA using liposomes according to Martin-Creuzburg et al. (2009). Three types of liposomes were prepared––cholesterol-containing liposome (“chol-liposome”), eicosapentaenoic acid (EPA)-containing liposome (“EPA-liposome”) and liposome without any substances added (“control-liposome”). Liposomes were prepared by adding 3.33 mg cholesterol or EPA to 3 mg 1-palmitoyl-2-oleoyl-phosphatidylglycerol, and 7 mg 1-palmitoyl-2-oleoyl-phosphatidylcholine dissolved in dichloromethane. Then, the resulting mixture was evaporated to dryness and suspended in 10 ml liposome buffer (20 mM Na_2_HPO_4_; 20 mM NaH_2_PO_4_; 150 mM NaCl, pH 7.0). Subsequently, the suspension was incubated on a rotary shaker (100 rpm) for 30 min and sonicated in an ultrasonic bath for 1 min. Then, suspensions were centrifuged (150,000*g*, 90 min, 4 °C), and afterward, the supernatant with an excess of free cholesterol or EPA was discarded and the pellet was resuspended in 10 ml liposome buffer. Aliquots of this liposome suspension were stored at −20 °C until use: liposomes were always sonicated for 2 min before being used.

### Analysis of fatty acids

Analyses of fatty acids were performed for two clones each of *D. longispina* (DlE and Dl4) and of *D. pulicaria* (DpBrA3 and DpBrS) and for four clones of *D. magna* (IL-M1-12, IL-M1-8, FI-N26-8c, and FI-N47-20). Analyses were carried out with neonates (<18 h old, average dwt per sample 489.55 ± 32.21 µg) from the second clutch of the clonal mothers and with 6-day-old animals at the end of the additional growth experiments (average dwt per sample 575.91 ± 31.85 µg). From the EPA-dependent functional response of each clone, we calculated the dietary EPA content that resulted in 75 % of the maximum growth rate, i.e., in a 25 % EPA limitation of growth (Table 4). Using this EPA concentration, we then ran additional growth experiments with each of the clones being exposed to the same relative degree of EPA limitation for 6 days and determined the increase in body mass and in the content of fatty acids per individual during those 6 days. These data allowed for the calculation of the EPA content of newly acquired biomass (minimum tissue quota for EPA), which we regarded as a measure of resource-use efficiency of the different *Daphnia* clones. The additional experiments were performed according to the same protocol as the previously described growth experiment.

For fatty acid analysis, *Daphnia* lipids were extracted twice with 8 ml dichloromethane/methanol (2:1, v:v) with prior addition of heptadecanoic acid methyl ester (C17:0 ME) and tricosanoic acid methyl ester (C23:0 ME) as an internal standard. The evaporated sample was transesterified with 5 ml of 3 N methanolic HCl at 70 °C for 20 min to yield fatty acid methyl esters (FAMEs) that were extracted with 3 × 2 ml iso-hexane. The hexane phase was subsequently evaporated to dryness and dissolved in 40–200 μl isohexane, of which 1 µl was subjected to gas chromatographic analysis on an 6890-N GC System (Agilent Technologies, Waldbronn, Germany) equipped with a DB-225 capillary column (30 m, 0.25 mm i.d., 0.25 µm film thickness, J&W Scientific, Folsom, CA, USA). The GC conditions were as follows: injector and FID temperatures 200 °C; initial oven temperature 60 °C for 1 min, followed by a 120 °C/min temperature ramp to 180 °C, then a ramp of 50 °C/min to 200 °C followed by 10.5 min at 200 °C, followed by ramp of 120 °C/min to 220 °C followed by 7.5 min at 220 °C; helium with a flow rate of 1.5 ml/min was used as the carrier gas. FAMEs were identified by comparing retention times with those of the reference compounds, and then quantified using the internal standard and previously established calibration functions for each individual FAME (von Elert 2002).

Total polyunsaturated fatty acids (total PUFA) is the sum of linoleic acid (18:2 ω-6), α-linolenic acid (ALA; 18:3 ω-3), eicosapentaenoic acid (EPA; 20:5 ω-3), and eicosatrienoic acid (ETE; 20:3 ω-3); other PUFAs were not detected in the samples. Total ω-3 PUFA content is the sum of α-linolenic acid (ALA; 18:3 ω-3), eicosapentaenoic acid (EPA; 20:5 ω-3), and eicosatrienoic acid (ETE; 20:3 ω-3); other ω-3 PUFAs were not detected in the samples. We normalized fatty acid compositions to dry weight (dwt) in neonates and 6-day-old animals of all clones and calculated mean values for the three species. Data for fatty acid content of *Daphnia* neonates (Table 5) are means ±1 SE with *n* = 3 (6 cases) and *n* = 2 (2 cases). Data for fatty acid content of 6-day-old animals (Table 6) are means ±1 SE with *n* = 3 (6 cases) and *n* = 2 (1 case) and *n* = 1 (1 case).

### Statistical analyses

Non-linear mixed models (Pinheiro and Bates 2000) based on a Monod-like saturation function (cf. Sperfeld and Wacker 2011) with an additional shift parameter *g*_0_ were applied to calculate the half-saturation constant *K*_S_ for growth as a function of the dietary EPA content

$$g = g_{0} + \frac{{(g_{\infty } - g_{0} ) \cdot S}}{{\left( {S + K_{\text{S}} } \right)}}$$

where *g*_0_ is the juvenile growth rate without EPA supplementation (day^−1^), *g*_∞_ is the asymptotic growth rate at saturating EPA concentration (day^−1^), *S* is the dietary concentration of EPA (μg EPA mg C^−1^), and *K*_S_ is the half-saturation constant (μg EPA mg C^−1^).

Two series of models were fitted to the data (Table 3): Models of type A assumed either no clonal differences (models A2 and A1) or a clonal random effect (model A3) for the half-saturation constant *K*_S_, whereas for all models of type B, the half-saturation parameter *K*_S_ was assumed to be dependent on size (*L*), i.e., *K*_S_ = *K*_0_ + *K*_L_*·L* with base level *K*_0_ and slope *K*_L_. This means that models of type A contained three parameters for the fixed effect (*g*_0_, *g*_∞_, *K*_S_) and models of type B four parameters (*g*_0_, *g*_∞_, *K*_0_, *K*_L_). Potential individual differences of the clones were expressed with up to three random-effect parameters (*g*_0_, *g*_∞_, *K*_S_), which were reduced by stepwise model selection. The second character of the model name (A1…A3, B1…B3) indicates the number of random effect parameters. Fixed effects describe the overall behavior, whereas random effects describe clonal differences. If a model includes both a fixed and a random effect for the same parameter, total values for each clone are their sum. To select an optimal model, AIC-based model selection was used (cf. Johnson and Omland 2004). Pairwise likelihood ratio tests were applied for model comparison between corresponding candidate models A and B. Standard errors were estimated by residual bootstrapping (cf. Efron and Tibshirani 1994).

The same technique was also applied to clutch size data (*c*). The data showed considerable scatter, the clones D.g. and D.h. did not develop eggs at all, and variance of the residuals showed dependence on the mean values. It was, therefore, necessary to omit the treatments without eggs and to apply a variance stabilizing transformation. Graphical analysis of the residuals showed that an arcsin square root function ($c' = 2\arcsin (\sqrt c )/\pi$ cf. Zar 1996) solved this problem.

Kruskal–Wallis non-parametric one-way analysis of variance was used to test the effect of species on fatty acid content in neonates and in 6-day-old *Daphnia*. The statistical analysis was performed using the R system for statistical computing (R Core Team 2015) and the add-on package lme4 (Bates et al. 2015a, 2015b) for the non-linear mixed models. All results are presented as a mean ±1 SE, and significance levels of all tests were set to *α* = 0.05. Text, figures, and tables refer to the parametric estimates, except stated otherwise.

## Results

**Fig. 1:**
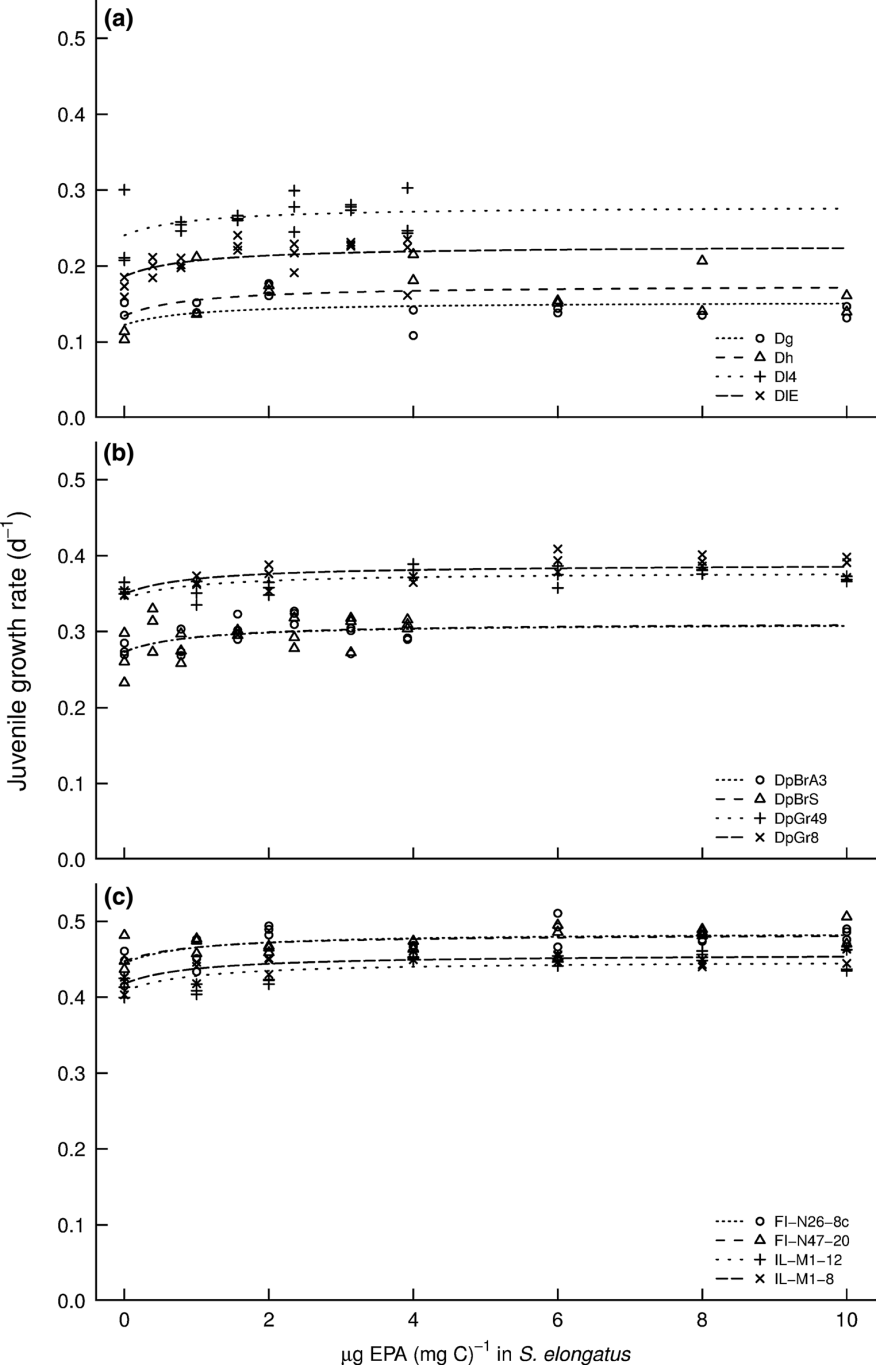
Juvenile growth rate at 20 °C and non-linear regression lines for *Daphnia* clones that had been fed with *S. elongatus* supplemented with cholesterol and increasing concentrations of EPA. The non-linear fits were obtained with the mixed model A3 where all parameters (*K*_S_, *g*_0_, and *g*_∞_) were allowed to vary randomly between clones: **a** small-sized *D. longispina* complex (*N* = 10), **b** medium-sized *D. pulicaria* (*N* = 10), and **c** large-sized *D. magna* (*N* = 5)

Based on regression lines, with increasing concentrations of eicosapentaenoic acid (EPA) in the food suspensions, juvenile growth rates increased for all the daphnids tested (Fig. 1a–c). Mean growth rates without EPA and chol-liposomes were 0.099 ± 0.033 day^−1^ for the *D. longispina* complex, 0.087 ± 0.004 day^−1^ for the *D. pulicaria* and 0.108 ± 0.010 day^−1^ for the *D. magna* (Table 2). Mean growth rates under optimal food conditions (control (1) with *C. klinobasis* as food; Table 2) were 0.288 ± 0.018 for the *D. longispina* complex, 0.395 ± 0.046 for *D. pulicaria* and 0.543 ± 0.006 for *D. magna*.

**Table 2:** Mean juvenile growth rates at 20 °C in the control treatment for *Daphnia* clones that had been fed with (1) the green algae *Chlamydomonas klinobasis*, (2) the cyanobacterium *Synechococcus elongatus* without any liposomes, and (3) *S. elongatus* with control liposomes of a concentration equivalent to the total concentration of cholesterol- and EPA-liposmoes added to the food treatment with 10 μg EPA mg C^−1^

Species	Clones	(1) *C. klinobasis*	(2) *S. elongatus* without any liposomes	(3) *S. elongatus* with control liposomes
*D. longispina* complex	DlE	0.268 ± 0.002	0.089 ± 0.029	0.196 ± 0.073
	Dl4	0.318 ± 0.004	0.028 ± 0.018	0.056 ± 0.029
	Dh	0.247 ± 0.003	0.078 ± 0.022	0.057 ± 0.012
	Dg	0.320 ± 0.032	0.092 ± 0.005	0.089 ± 0.007
*D. pulicaria*	DpBrA3	0.288 ± 0.008	0.107 ± 0.008	0.099 ± 0.019
	DpBrS	0.347 ± 0.010	0.135 ± 0.026	0.084 ± 0.011
	DpGr8	0.460 ± 0.006	0.062 ± 0.012	0.088 ± 0.053
	DpGr49	0.484 ± 0.010	0.057 ± 0.004	0.079 ± 0.006
*D. magna*	IL-M1-12	0.534 ± 0.008	0.102 ± 0.004	0.087 ± 0.013
	IL-M1-8	0.542 ± 0.002	0.118 ± 0.005	0.132 ± 0.003
	FI-N26-8c	0.559 ± 0.014	0.137 ± 0.008	0.114 ± 0.010
	FI-N47-20	0.537 ± 0.014	0.088 ± 0.012	0.099 ± 0.009

Depicted are mean juvenile growth rates for four clones each of three *Daphnia* species: the small-sized *D. longispina* complex, the medium-sized *D. pulicaria,* and the large-sized *D. magna*

Data are means ±1 SE with *n* = 3

**Fig. 2:**
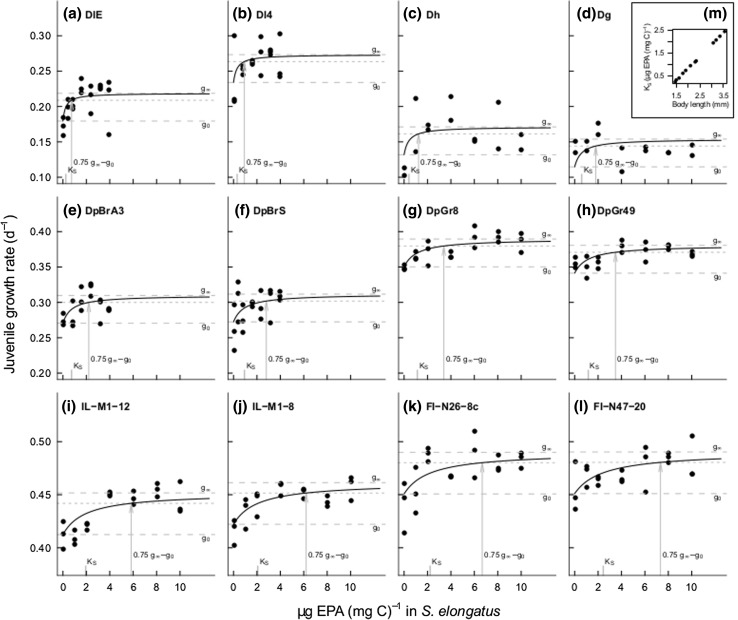
Juvenile growth rate at 20 °C and non-linear regression lines for *Daphnia* that had been fed with *S. elongatus* supplemented with cholesterol and increasing concentrations of EPA (*N* = 10 **a**–**h** and *N* = 5 **i**–**l**). The non-linear fits were obtained with the mixed model B1 with *a clonal* random effect for *g*_0_ and a body size-dependent half-saturation constant *K*_S_, see *inset* figure (*m*). Note different scaling of *Y*-axis between the three species (*rows*)

To test whether *K*_S_ is body size-dependent, we compared two series of non-linear mixed models, with models of type A assuming none or a random effect for *K*_S_, i.e., that the clones do not differ or that they differ in a non-systematic way, respectively, and models of type B assuming a fixed clonal effect, i.e., that the half-saturation constant *K*_S_ is linearly body size-dependent with *K*_S_ = *K*_0_ + *K*_L_·*L*. Model selection revealed that models of type B fitted better to the data (larger log-likelihood) and that model B1 was the optimal model because of its lowest Akaike information criterion (AIC). Pairwise model comparisons confirmed that all models of type B were significantly better than the corresponding models of type A (likelihood ratio test, cf. Table 3). In conclusion, models of type B had a higher explanatory value for the dependency of juvenile somatic growth rates on dietary EPA concentrations than models of type A. In contrast to models of type A, models of type B assumed a body size dependency of *K*_S_, and thus, the results show that the half-saturation constant *K*_S_ is body size-dependent, with lowest values for the small-bodied species (Tables 3, 4). The half-saturation constant *K*_S_, which indicates the EPA concentration for 50 % growth saturation, increased significantly with increasing body size of the tested animals (model B, Table 4; Fig. 2).

**Table 3:** Model selection and likelihood ratio tests of non-linear mixed models based on a Monod-like saturation function

Model	Model parameters		*Df*	AIC	LogLik	Chisq	Chi *Df*	*p* value
	Fixed effect	Clonal random						
A3	*g*_0_, *g*_∞_, *K*_S_	*g*_0_, *g*_∞_, *K*_S_	7	−1116.5	565.3	5.73	1	0.017
B3	*g*_0_ *, g*_∞_, *K*_0_, *K*_L_	*g*_0_, *g*_∞_, *K*_S_	8	−1120.2	568.1			
A2	*g*_0_, *g*_∞_, *K*_S_	*g*_0_, *g*_∞_	6	−1118.5	565.3	5.73	1	0.017
B2	*g*_0_, *g*_∞_, *K*_0_, *K*_L_	*g*_0_, *g*_∞_	7	−1122.2	568.1			
A1	*g*_0_, *g*_∞_, *K*_S_	*g*_0_	5	−1120.3	565.2	5.27	1	0.022
B1	*g*_0_, *g*_∞_, *K*_0_, *K*_L_	*g*_0_	6	−1123.6	567.8			

Model type A: common half-saturation constant for all clones (models A1, A2) or non-systematically varying due to a clonal random effect (A3), model type B: size dependency of *K*_S_ = *K*_0_ + *K*_L_ *·L*

*Df* degrees of freedom of the model, *AIC* Akaike information criterion, *LogLik* logarithm of maximum likelihood

The *p*-values indicate that models with size-dependent *K*_S_ (models B) are significantly superior to their corresponding model of type A (Chi-square likelihood ratio test)

**Table 4:** Half-saturation constant *K*_S_ calculated with the non-linear mixed model B1 based on a Monod-like saturation function, for four clones each of three *Daphnia* species: the small-sized *D. longispina* complex, the medium-sized *D. pulicaria,* and the large-sized *D. magna*

Species	Clones	*L*	SE	Half-saturation constant K_S_ (μg EPA mg C^−1^)	SE	Concentration of EPA resulting in 75 % of asymptotic growth rate (μg EPA mg C^−1^)	SE
*D. longispina* complex	DlE	1.43	0.02	0.25 (0.39)	(0.50)	0.74 (1.17)	(1.51)
	Dl4	1.49	0.02	0.31 (0.45)	(0.48)	0.93 (1.35)	(1.45)
	Dh	1.59	0.02	0.41 (0.55)	(0.46)	1.24 (1.66)	(1.39)
	Dg	1.77	0.02	0.60 (0.74)	(0.46)	1.80 (2.21)	(1.39)
*D. pulicaria*	DpBrA3	1.90	0.01	0.74 (0.87)	(0.49)	2.21 (2.61)	(1.48)
	DpBrS	2.09	0.03	0.93 (1.06)	(0.57)	2.80 (3.19)	(1.72)
	DpGr8	2.28	0.04	1.13 (1.26)	(0.68)	3.39 (3.77)	(2.04)
	DpGr49	2.31	0.03	1.16 (1.29)	(0.70)	3.49 (3.87)	(2.10)
*D. magna*	IL-M1-12	3.06	0.02	1.94 (2.06)	(1.25)	5.83 (6.53)	(3.74)
	IL-M1-8	3.18	0.02	2.07 (2.18)	(1.34)	6.21 (6.99)	(4.02)
	FI-N26-8c	3.33	0.09	2.22 (2.33)	(1.46)	6.67 (7.64)	(4.38)
	FI-N47-20	3.54	0.04	2.44 (2.55)	(1.63)	7.33 (7.48)	(4.89)

Depicted are concentrations of eicosapentaenoic acid (EPA) resulting in 75 % of asymptotic growth rate related to the baseline (*g*_∞_−*g*_0_)

*L* body size at first reproduction, *SE* standard error

Bootstrap estimates and standard errors are given in *parentheses*

We applied the optimal model B1 to estimate the dietary EPA-concentration at which saturation of growth occurred. The resulting regression lines showed that all clones of the small-bodied *D. longispina* species complex (Fig. 2a–d) reached saturation at a lower EPA concentration than the medium-bodied *D. pulicaria* (Fig. 2e–h), which had a lower saturating EPA concentration than the large-bodied *D. magna* (Fig. 2i–l). However, the juvenile growth rate increased with increasing body size (Fig. 1a–c): Already in the absence of EPA from the food, the large-bodied species grew faster than the small-bodied ones, with a mean juvenile growth rate of 0.432 ± 0.007 day^−1^ for *D. magna*, 0.312 ± 0.013 day^−1^ for *D. pulicaria* and 0.174 ± 0.018 day^−1^ for the *D. longispina* complex (Fig. 1a–c). Accordingly, the baseline growth rate parameter *g*_0_ correlated significantly with body size *L* in both models A1 and model B1 (Pearsons *r* = 0.91 and 0.92, respectively; *p* < 0.001, Tables A1, A2).

Mean clutch size slightly increased with body size and was 1.31 ± 0.29 for small-bodied *D. longispina,* 1.35 ± 0.23 for *D. pulicaria* and 2.48 ± 0.18 for *D. magna*. The results of the model selection for clutch size (*c*) were similar to the results of growth rate. Again, all models of type B with size-dependent half-saturation showed smaller AIC values and were significantly superior to their corresponding models of type A (Table A4). Model B1 was now both, the optimal model measured by AIC and one of the models with highest log likelihood. This means that EPA dependency of clutch size, similar as for juvenile growth rate, is higher for the large-bodied clones, whereas smaller clones showed lower half-saturation constants.

The observed size dependency of *K*_S_ for EPA-dependent juvenile growth might have been caused by species-specific differences in the fatty acid content of neonates, as higher maternal transfer of EPA into the eggs might result in a lower *K*_S_ for EPA-dependent juvenile growth. The mean total fatty acid content of neonates was 39.06 ± 3.39 ng µg^−1^ dwt and did not differ between species (Table 5, Kruskal–Wallis, *p* = 0.84). As we were considering EPA-dependent juvenile growth, and as EPA is an ω-3 polyunsaturated fatty acid (PUFA), we determined the mean total PUFA content to be 18.30 ± 2.01 ng µg^−1^ dwt, with no differences among species (Table 5, Kruskal–Wallis, *p* = 0.11). The picture did not change when considering only ω-3 PUFAs, of which the mean content was 17.07 ± 1.94 ng µg^−1^ dwt (Table 5, Kruskal–Wallis, *p* = 0.11). The mean eicosapentaenoic acid (EPA) content of neonates was 0.44 ± 0.12 ng µg^−1^ dwt and did not differ significantly between species (Table 5, Kruskal–Wallis, *p* = 0.06). In conclusion, the fatty acid content of neonates could not explain the observed size dependency of *K*_S_ for EPA-dependent juvenile growth.

**Table 5:** Fatty acid content of *Daphnia* neonates (<18 h old) of two clones of the small-sized *D. longispina*, two clones of the medium-sized *D. pulicaria,* and four clones of the large-sized *D. magna*

Species	Clones	Total FA (ng μg^−1^ dwt)	Total PUFA (ng μg^−1^ dwt)	Total ω-3 PUFA (ng μg^−1^ dwt)	Total EPA (ng μg^−1^ dwt)
*D. longispina*	DlE	47.36 ± 5.96	25.53 ± 4.13	24.81 ± 3.52	1.55 ± 0.24
	Dl4	35.80 ± 7.85	19.24 ± 6.50	17.79 ± 6.24	0.36 ± 0.24
	Mean	41.58 ± 5.78	22.39 ± 3.14	21.30 ± 3.51	1.07 ± 0.33
*D. pulicaria*	DpBrS	37.42 ± 8.23	14.91 ± 5.01	13.48 ± 4.80	0.06 ± 0.03
	DpBrA3	43.35 ± 17.39	9.76 ± 3.27	8.88 ± 3.07	0.38 ± 0.13
	Mean	40.39 ± 2.97	12.34 ± 2.58	11.18 ± 2.30	0.22 ± 0.09
*D. magna*	IL-M1-12	37.76 ± 1.94	20.35 ± 1.40	18.89 ± 1.36	0.34 ± 0.00
	IL-M1-8	37.26 ± 5.85	17.52 ± 1.88	16.11 ± 1.84	0.11 ± 0.04
	FI-N26-8c	50.16 ± 7.15	29.68 ± 4.61	27.97 ± 4.47	0.23 ± 0.07
	FI-N47-20	14.81 ± 8.27	5.99 ± 3.94	5.32 ± 3.60	0.35 ± 0.06
	Mean	35.00 ± 7.36	18.38 ± 4.88	17.07 ± 4.67	0.23 ± 0.04

Depicted are the content of total fatty acids (FA), total polyunsaturated fatty acids (PUFA), total ω-3 PUFA, and eicosapentaenoic acid (EPA)

Data are means ±1SE with *n* = 3 (6 cases) and *n* = 2 (2 cases)

Total PUFA is the sum of linoleic acid (18:2 ω-6), α-linolenic acid (ALA; 18:3 ω-3), eicosapentaenoic acid (EPA; 20:5 ω-3), and eicosatrienoic acid (ETE; 20:3 ω-3). Total ω-3 PUFA content is the sum of α-linolenic acid (ALA; 18:3 ω-3), eicosapentaenoic acid (EPA; 20:5 ω-3), and eicosatrienoic acid (ETE; 20:3 ω-3)

Alternatively, differences in *K*_S_ for EPA-dependent juvenile growth might be caused by differences in resource-use efficiency. In *Daphnia* that had been exposed to 75 % EPA saturation, the mean total fatty acid content of newly built biomass ranged from 9.23 to 99.55 ng µg^−1^ dwt and was significantly different among the three species (Table 6, Kruskal–Wallis, *p* = 0.017). The lowest fatty acid content in newly built biomass was observed in the small-bodied *D. longispina*, and the highest content in medium-bodied *D. pulicaria* (Table 6). The mean total PUFA content of newly built biomass ranged from 0.96 to 5.53 ng µg^−1^ dwt and did not differ among species (Table 6, Kruskal–Wallis, *p* = 0.40). The same was true for the mean content of ω-3 PUFAs, which ranged from 1.01 to 5.03 ng µg^−1^ dwt (Table 6, Kruskal–Wallis, *p* = 0.59). The mean EPA content of newly built biomass ranged from 0.16 to 1.86 ng µg^−1^ dwt and did not differ among species (Table 6, Kruskal–Wallis, *p* = 0.07).

**Table 6:** Fatty acid content of newly built biomass in *Daphnia* that had been fed with *S. elongatus* supplemented with cholesterol and a concentration of eicosapentaenoic acid (EPA) for 6 days, resulting in 75 % of asymptotic growth rate (see Table 4)

Species	Clones	Total FA (ng μg^−1^ dwt)	Total PUFA (ng μg^−1^ dwt)	Total ω-3 PUFA (ng μg^−1^ dwt)	Total EPA (ng μg^−1^ dwt)
*D. longispina*	DlE	9.23 ± 0.00	nd	nd	nd
	Dl4	52.56 ± 33.68	5.53 ± 3.92	5.03 ± 3.65	0.78 ± 0.30
	Mean	38.12 ± 24.22	–	–	–
*D. pulicaria*	DpBrS	79.46 ± 11.30	0.96 ± 0.21	1.01 ± 0.22	0.25 ± 0.12
	DpBrA3	99.55 ± 8.48	2.59 ± 0.58	2.20 ± 0.57	0.16 ± 0.02
	Mean	89.51 ± 7.75	1.77 ± 0.46	1.61 ± 0.38	0.20 ± 0.06
*D. magna*	IL-M1-12	27.51 ± 6.28	1.71 ± 0.09	1.34 ± 0.05	0.86 ± 0.18
	IL-M1-8	47.99 ± 13.08	2.45 ± 0.78	2.08 ± 0.74	1.22 ± 0.50
	FI-N26-8c	69.43 ± 5.44	2.64 ± 1.44	1.42 ± 0.95	1.86 ± 1.16
	FI-N47-20	64.28 ± 3.37	2.21 ± 0.04	1.82 ± 0.05	0.24 ± 0.07
	Mean	52.30 ± 5.98	2.22 ± 0.29	1.67 ± 0.27	1.05 ± 0.33

Depicted are the content of total fatty acids (FA), total polyunsaturated fatty acids (PUFA), total ω-3 PUFAs, and eicosapentaenoic acid (EPA) for two clones of the small-sized *D. longispina*, two clones of the medium-sized *D. pulicaria,* and four clones of the large-sized *D. magna*

Data are means ±1 SE with *n* = 3 (6 cases) and *n* = 2 (1 case) and *n* = 1 (1 case)

Total PUFA is the sum of linoleic acid (18:2 ω-6), α-linolenic acid (ALA; 18:3 ω-3), eicosapentaenoic acid (EPA; 20:5 ω-3), and eicosatrienoic acid (ETE; 20:3 ω-3). Total ω-3 PUFA content is the sum of α-linolenic acid (ALA; 18:3 ω-3), eicosapentaenoic acid (EPA; 20:5 ω-3), and eicosatrienoic acid (ETE; 20:3 ω-3)

*nd* not detected

## Discussion

Our results show that the clones of the small-bodied *D. longispina* complex reach saturation at lower EPA concentrations than the medium-bodied *D. pulicaria* and the large-bodied *D. magna*. The half-saturation constant *K*_S_ increased significantly with increasing body size of the tested species. This finding that small-bodied species have a lower EPA threshold corroborates other published data about single clones of different species: For the medium-bodied *D. pulex,* a lower EPA threshold for 90 % saturation––1.3 μg EPA mg C^−1^ (Ravet et al. 2012)––has been reported than for the large-bodied *D. magna* (2.0–4.9 μg EPA mg C^−1^; Sperfeld and Wacker 2011). Body size dependence of the half-saturation constant for clutch size points in the same direction, although large-bodied species had a higher clutch size (Bengtsson 1986).

Taking into account that the PUFA content in algae is negatively correlated with temperature (Renaud et al. 1995; Fuschino et al. 2011), lower EPA thresholds may promote small-bodied species in warmer environments. This has experimentally been tested by Sikora et al. (2014): when a green alga was grown at different temperatures, temperature was inversely related to the content of ω-3 PUFAs in the food. When this alga was fed to *Daphnia*, the algal food quality decreased with the temperature of the culture, and this negative effect of temperature on growth was more pronounced in the large-sized *D. pulicaria* than in the small-bodied *D. cucullata*. Furthermore, the decrease in the content of EPA in natural phytoplankton with increased temperature is caused by an increased relative abundance of cyanobacteria, which in general do not contain PUFAs (Martin-Creuzburg et al. 2008; Paerl and Huisman 2008; Soares et al. 2013). In conclusion, the lower thresholds for EPA-saturated growth for small-bodied species and the negative relation of dietary EPA content with ambient temperature in phytoplankton should promote small-bodied species in warm environments. This suggestion is in line with the observed global distribution of cladocerans, which is characterized by a frequent predominance of small-bodied cladoceran taxa in tropical and subtropical waters (Atkinson 1994; Gillooly and Dodson 2000).

Also noteworthy is that the number of large-bodied species decreases in temperate lakes during summer, a phenomenon known as ‘midsummer decline’ (Sommer et al. 1986). This phenomenon could be explained by various factors mainly predation and quantity and quality of food (Wagner et al. 2004). One aspect of food quality is the presence of filamentous cyanobacteria that have been shown to promote small bodied species due to their lower vulnerability to feeding interference caused by the filaments (Gliwicz and Lampert 1990). As suggested by Müller-Navarra et al. (2000), the poor quality of cyanobacterial food is as well due to the lack of PUFAs. In this context, the higher EPA thresholds of large-bodied *Daphnia* provide another mechanism that contributes to the ‘midsummer decline’ of large-bodied zooplankton. It is even possible that the low biochemical quality of cyanobacterial food has a stronger negative effect on the competitive ability of cladocerans than mechanical interference (Kurmayer 2001).

The lower EPA thresholds of the small-bodied *D. longispina* complex reported here could not be explained by differences in the fatty acid content of neonates, as the fatty acid content did not differ among neonates of different species.

Many planktonic primary producers can store nutrients during periods of high nutrient availability that allow for higher growth rates than predicted by the Monod equation during subsequent periods of low nutrient availability. In these cases, growth is a function of the nutrient content in the cell, and competition is determined by the minimum cell nutrient content, the cell quota (Droop 1983). Both storage and maternal transfer have been demonstrated for fatty acids and for EPA (Cowgill et al. 1984; Becker and Boersma 2005; Wacker and Martin-Creuzburg 2007; Sperfeld and Wacker 2015), so that, similar to nutrients in phytoplankton, during EPA limitation, a tissue quota of EPA might determine competition among differently sized *Daphnia* species. We, therefore, grew all clones at the same degree of EPA limitation and determined the minimum content of EPA in newly built biomass. No differences in the content of EPA in newly built biomass were detectable between the species. However, the resource-use efficiency might more appropriately be determined by measuring amount of EPA ingested and newly built biomass achieved by ingested EPA.

Alternatively, it is also possible that EPA growth saturation thresholds are not a body size-related trait, but are rather the result of adaptation to the local environment. Brzeziński and von Elert (2007) showed differences in sensitivity to the absence of EPA among clones of species with similar body size (*D. galeata*, D*. hyalina,* and their hybrids), which suggest genetic adaptation to environmental heterogeneity. The EPA threshold may vary with food type (cyanobacterium or the green algae), which suggests that some of the food components (e.g., phosphorus, other fatty acids, and amino acids) influence the EPA threshold (Becker and Boersma 2010; Ravet et al. 2012). DeMott and Müller-Navarra (1997) supposed that the higher growth rates of *D. magna* compared with both *D. galeata* and *D. pulicaria* fed with *S. elongatus* resulted from the adaptation of the former species to pond environments, which often lack PUFA-rich algae. However, our findings that (1) EPA demands are inversely related to size and that (2) the content of EPA in newly built biomass did not differ among species, and do not support the reasoning of DeMott and Müller-Navarra (1997).

The growth rates obtained in treatments with no supplementation of EPA were considerably higher than reported in several other papers (DeMott and Müller-Navarra 1997; DeMott 1998). These high growth rates are due to the fact that here the treatments were supplemented with cholesterol to account for the general absence of this essential lipid from cyanobacteria (von Elert et al. 2003) and are in close accordance with the results from other experiments, in which cyanobacteria were supplemented with cholesterol (e.g., Martin-Creuzburg et al. 2008, 2009). We have found that juvenile growth rates with only cholesterol supplementation increased with body size. Large-bodied species grew faster, even in the absence of EPA.

We have measured mass-specific growth rates and clutch size, and their value for making predictions for competition is very limited. To draw robust conclusions about competition would require other approaches, e.g., measuring population growth rates (e.g., instantaneous rate of increase) of species either in isolation followed by competition models (e.g., resource-ratio theory of competition, Tilman 1982) or by keeping more than one species together in a competition arena (e.g., Sikora and Dawidowicz 2014). Differences in the requirement of PUFAs in *Daphnia* species of various body sizes may influence interspecific competition (DeMott 1989), i.e., species with lower requirements should be better competitors, especially when fatty acids are limiting for *Daphnia* growth and reproduction. The concentration of EPA in lake seston may vary seasonally in the range of 0.2–20.6 μg mg POC^−1^, with the lowest concentration during summer months (Müller-Navarra et al. 2000; Gladyshev et al. 2011; Sperfeld and Wacker 2011). Assuming a saturation threshold for EPA-limited growth of *Daphnia*, Ravet et al. (2012) estimated that an EPA limitation of *Daphnia* growth occurs to a varying extent in 40–87 % of the lakes. Our data indicate that the higher juvenile growth rate of the large-bodied species can override the higher EPA threshold resulting presumably in a competitive superiority of large-bodied species. However, the size dependency of saturation threshold for EPA-dependent growth provides a new mechanistic explanation for the dominance of small-bodied species in warmer environments and its prevalence at low latitudes. In addition, these differences in EPA-dependent growth saturation may contribute to the ‘midsummer decline’ of large-bodied zooplankton.
